# Health-related quality of life and hospital costs of Finnish melanoma patients participating in the second Multicenter Selective Lymphadenectomy Trial

**DOI:** 10.2340/1651-226X.2025.42314

**Published:** 2025-02-10

**Authors:** Pia J. Heino, Jukka Pappinen, John F. Thompson, Micaela M. Herberg, Tiina A. Jahkola, Mark B. Faries

**Affiliations:** a Department of Plastic Surgery, University of Helsinki and Helsinki University Hospital, Helsinki, Finland; b Faculty of Medicine, Department of Public Health, University of Helsinki, Helsinki, Finland; c The University of Sydney, Sydney, Australia; d Comprehensive Cancer Center, Helsinki University Hospital and University of Helsinki, Helsinki, Finland; e Cedars-Sinai Medical Center, Los Angeles, USA; f The Angeles Clinic & Research Institute, Los Angeles, USA

**Keywords:** Melanoma, cutaneous, costs and cost analysis, quality of life, lymph node excision, sentinel lymph node biopsy, clinical trial

## Abstract

**Background and purpose:**

After reports that complete lymph node dissection (CLND) did not improve melanoma-specific survival of sentinel lymph node (SLN)-positive patients, the use of CLND has diminished but it is still carried out for selected patients. We sought to assess differences in Health-Related Quality of Life (HRQoL) and tertiary care costs among the Finnish Multicenter Selective Lymphadenectomy Trial (MSLT)-II-patients.

**Patients/materials and methods:**

A total of 52 patients randomized to CLND and 55 to nodal observation completed a modified version of the standardized and validated, RAND-36 questionnaire at baseline, 4 months and annually up to 5 years. Tertiary care costs between the groups were also compared.

**Results:**

At 60 months, the mean HRQoL score for the CLND and observation groups for General Health were 77.3 versus 65.0 (*p* = 0.007, adjusted *p* = 0.065), for role limitations due to physical health 89.5 versus 72.3 (*p* = 0.029, adjusted *p* = 0.203) and for role limitations due to emotional problems 91.4 versus 71.9 (*p* = 0.006, adjusted *p* = 0.065) and at 48 months, 92.8 versus 71.3 (*p* = 0.002, adjusted *p* = 0.056). Median costs per patient were higher in the CLND group at 4 months but the difference disappeared during follow-up.

**Interpretation:**

This study suggests that undergoing CLND after a positive SLN biopsy is not a predictor of worse HRQoL. CLND generates greater costs initially, but there seem to be no major differences in total cost per patient between the two groups.

## Introduction

Two landmark trials, the second Multicenter Selective Lymphadenectomy Trial (MSLT-II) [1], and German Cooperative Oncology Group Selective Lymphadenectomy Trial (DeCOG-SLT) [2], found no survival benefit from performing immediate completion lymph-node dissection (CLND) among patients with a positive sentinel lymph node biopsy (SLNB) in comparison to nodal observation. As a result, the use of CLND in SLN-positive melanoma patients has diminished greatly over recent years [3, 4]. However, CLND is still carried out for patients who cannot readily be followed up for nodal recurrence or who are unfit for adjuvant systemic therapy, to reduce the likelihood of missed nodal recurrence and achieve better local disease control. While it is widely accepted that CLND is associated with a higher risk of short-term and long-term morbidity including lymphedema than SLNB alone [1, 5–7], there are few existing studies examining how performing immediate CLND versus nodal observation affects Health-Related Quality of Life (HRQoL) [8, 9]. Our aim was to assess differences in HRQoL among the Finnish cohort of MSLT-II -patients. While HRQoL is an important outcome measure in randomized controlled trials, it has become increasingly important to simultaneously analyze health-care costs to obtain information on how to allocate health-care resources between diseases and therapies. Thus, as a secondary outcome, we compared melanoma-related health-care costs for the two randomized groups.

### Patients/materials and methods

The MSLT-II patient selection process and study design have been fully described previously [1]. In Finland, the study was carried out at the Plastic Surgery Department of the Helsinki University Hospital. Patients with a positive SLNB were randomized to CLND (*n* = 55) or nodal observation (*n* = 55) between June 2006 and February 2014. All provided written informed consent. Three patients randomized to CLND declined surgery and were excluded; thus the final CLND group comprised 52 patients, all of whom completed a 28-item Health -Related Quality of Life survey (Medical Outcomes Study [MOS] Health survey, see Supplementary Appendix), which is a hybrid questionnaire based on the MOS Core Measures of HRQoL [10] and RAND-36 [11]. Questions 1–5c correspond to items 1–19 and questions 6a-c to items 33–36 in the standardized and validated, generic RAND 36-Item Health Survey 1.0, which has 36 items identical to the 36-Item Short Form Health Survey (SF-36) [12]. Questions 7a-e correspond to questions 30, 33, 24, 53 and 54 of the MOS Core Survey [10], respectively (see Appendix). The questionnaires were completed at baseline (before randomization) and at 4, 12, 24, 36, 48 and 60 months. RAND-36-subscales General health, Physical functioning, Role limitations due to physical health and Role limitations due to emotional problems were determined from the answers to the HRQoL questionnaire. The answers to questions 1–5c and 6a–c were scored as instructed by RAND Health Care [13]. A difference of five points or more on a 0–100 scale was considered clinically meaningful [11]. Subscale scores were calculated if participants answered at least half of the items in the subscale. Missing items within a subscale were excluded from scoring, and the mean of the available items was used to calculate the subscale score. Participants with fewer than half of items answered in a subscale were excluded from subscale-level analyses. The HRQoL questionnaire used in MSLT-II did not contain RAND-36-compatible questions related to Energy/fatigue, Social Functioning, Pain, Emotional well-being or Health change, thus we were unable to get a fully validated HRQoL index.

In addition, patients completed a 47–item quality of life survey (see Appendix) designed to capture physical functioning, symptoms, emotional well-being and overall quality of life. This survey included the question ‘Have you noticed swelling on your body?’. Patients were asked to write the body site where they had experienced swelling. If a patient who had either axillary or groin SLNB or CLND reported swelling in the limb of the operated side, it was recorded. Because the 47-item quality of life-survey has not yet been standardized and validated, the results were not analyzed for this study and only information about patients’ perceptions about swelling in the operated limb was utilized.

In both groups, patients underwent clinical follow-up every 4 months for 2 years and every 6 months thereafter up to 5 years. In addition, the observation group patients were followed up with ultrasound. If nodal recurrence without distant metastasis was detected, the patient underwent therapeutic lymph node dissection. Most patients in each group were also followed up with CT-scans every 6 months during the first 2 years and annually thereafter up to 5 years. At follow-up visits the presence of lymphedema was assessed by a clinician. They assessed swelling in the operated extremity or asymmetry in limb circumference.

Data documenting melanoma-related tertiary care health-care costs were retrieved from the hospital registry. To calculate and compare melanoma-related costs between the two groups during the 5-year follow-up period, cost data were retrieved by examining melanoma-related DRG products and their invoice data generated in tertiary health care for each patient during the follow-up period. This was done by retrieving all costs related to the ICD-10-diagnosis code C43 (cutaneous melanoma). For the CLND group, cost data were available only from 1 January 2009 onwards. For the nodal observation group, cost data excluding outpatient clinic and ultrasound follow-up costs were missing before 2009. Missing costs generated between June 2006 and December 2008 were imputed by using the mean and median costs derived from cost data available from 1 January 2009 onwards for the whole group (CLND or nodal observation). To calculate the distribution of costs and the costs of complications for the two groups we used cost data available from 1 January 2009 to 31 December 2019. Each HRQoL subscale was compared separately at individual timepoints with the Mann–Whitney-*U*-test. We investigated the potential interdependence between the four subscales using Spearman correlation tests. Statistical significance was set at *p* < 0.05, with adjustments for multiple comparisons made using the Benjamini–Hochberg False Discovery Rate (FDR) correction. Statistical analysis was carried out using IBM SPSS Statistics versions 26 and 29.

## Results

Study population characteristics are presented in Table 1. There were no statistically- significant differences between the two groups in sex, age, Breslow thickness, primary excision site, complications at primary excision or SLNB sites, adjuvant therapies or operations for metastatic disease, or systemic therapy for metastatic disease. The cumulative lymphedema rates during the 5-year follow-up period in the CLND and nodal observation groups were 65.4% and 32.7%), respectively (*p* < 0.001). The lymphedema prevalence and cumulative incidence are presented in Table 1.

**Table 1:** Study population characteristics.

Characteristic	Dissection, *n* = 52 *n* (%)	Nodal observation, *n* = 55 *n* (%)	*p*
Sex			0.847**
female n:o (%)	29 (55.8)	29 (52.7)	
male n:o (%)	23 (44.2)	26 (47.3)	
Age – years			0.091#
mean (SD)	55.8 (10.1)	53.8 (12.8)	
median (range)	57 (29–73)	55 (24–75)	
Breslow thickness			0.783#
mean (SD)	2.8 (1.8)	2.6 (1.8)	
median (range)	2.1 (0.7–9.0)	2.0 (0.8–9.0)	
Primary site n:o (%)			0.438*
trunk	23 (44.2)	25 (45.5)	
lower extremity	21 (40.4)	19 (34.5)	
upper extremity	3 (5.8)	8 (14.5)	
head & neck	5 (9.6)	3 (5.5)	
complication in primary excision site n:o (%)			0.113*
Clavien Dindo (n:o)	14 (26.9)	8 (14.5)	
I	7	6	
II	4	1	
IIIa	1	1	
IIIb	2		
complication in SNB site n:o (%)			
Clavien -Dindo (n:o)	14 (26.9)	16 (29.1)	0.803*
I	6	14	
II	7	2	
IIIb	1		
complication in CLND site n:o (%)			
Clavien-Dindo Class	25 (48.1)		
I	17		
II	7		
IIIb	1		
CLND localization			
axilla	24 (43.6		
groin	23 (41.8)		
neck	5 (9.1)		
Received adjuvant interferon	6 (11.5)	5 (9.1)	0.677*
Received oncological therapy for metastatic disease during 5 year follow-up	12 (21.2)	11 (20.0)	0.696*
Received radiotherapy during 5 year follow up	11	12	0.982*
Were operated for metastasis during 5 year follow up	3	4	0.532*
Lymphedema prevalence (months)			
4	22 (44.9)	11 (20.3)	0.007**
12	20 (42.5)	7 (14.0)	0.008**
24	17 (38.6)	5 (11.1)	0.025**
36	13 (29.5)	3 (6.9)	0.034**
48	10 (22.7)	5 (11.6)	0.118**
60	7 (20.0)	5 (11.6)	0.717**
Cumulative lymphedema incidence rate (months)			
4	22 (42.3)	11 (20.0)	0.007**
12	27 (52.9)	14 (25.5)	0.003**
24	31 (59.6)	15 (27.3)	<0.001**
36	32 (61.5)	16 (27.3)	<0.001**
48	34 (65.4)	17 (30.9)	0.002**
60	34 (65.4)	18 (34.6)	<0.001*
Axillary or groin dissection patients reporting any ‘A little’, ‘Quite a bit’ or ‘Very’ swelling in the limb of the operated side at			
Baseline (months)	10 (23.3)	8 (16.7)	0.045**
4	26 (65)	13 (27.7)	< 0.001**
12	21 (55.3)	11 (32.4)	0.006 **
24	22 (61.1)	6 (17.1)	< 0.001*
36	14 (48.3)	6 (17.6)	0.014**
48	14 (48.3)	6 (18.8)	0.028**
60	11 (40.7)	6 (18.8)	0.086**
Number of non-respondents/alive subjects (%) (months)			
Baseline	0/52 (0)	0/55 (0)	0.673**
4	3/52 (5.8)	2/55 (3.6)	0.758**
12	6/50 (12.0)	5/52 (9.6)	0.488**
24	3/46 (6.5)	6/49 (12.2)	0.360**
36	9/46 (19.6)	5/47 (10.6)	1.000**
48	5/42 (11.9)	5/47 (10.6)	0.417**
60	10/41 (24.4)	7/45 (15.6)	
Observation group: underwent CLND during follow-up n (%)		17 (15.9)	
Observation group average follow-up time until CLND in months:			
mean (SD)		24.5 (19.9)	
median (range)		13.0 (2.0–59.0)	

* X^2^ test

** Fisher’s exact test

# independet samples T-test

The proportion of non-respondents, defined as patients who left the whole questionnaire unanswered, did not differ between the CLND and observation groups (Table 1). The non-respondents did not differ from the respondents by age, surgical complications, metastatic disease or baseline HRQoL in any of the four subscales. However, there was a significant association between response behavior and survival status at 5 years (*p* = 0.028). A total of 77.9% (*n* = 53) of those who completed all questionnaires were alive at 5 years versus 56.4% (*n* = 22) of those who left at least one questionnaire unanswered during follow-up.

### Health–related quality of life

**Figure 1:**
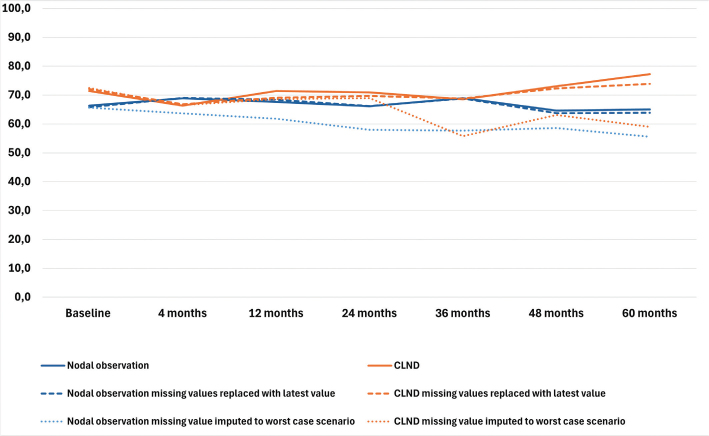
General health mean scores.

**Figure 2:**
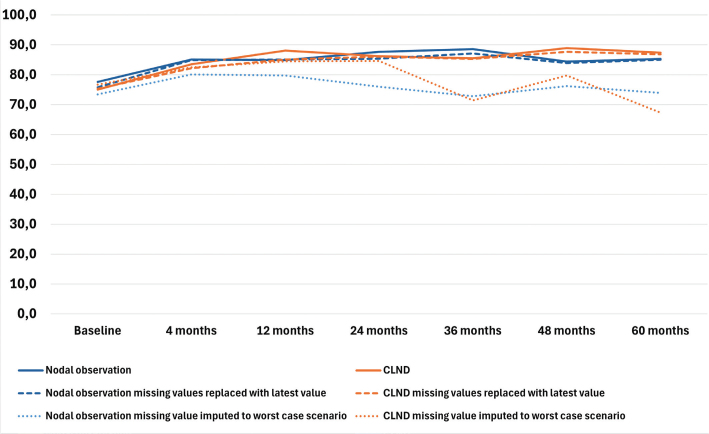
Physical functioning mean scores.

**Figure 3:**
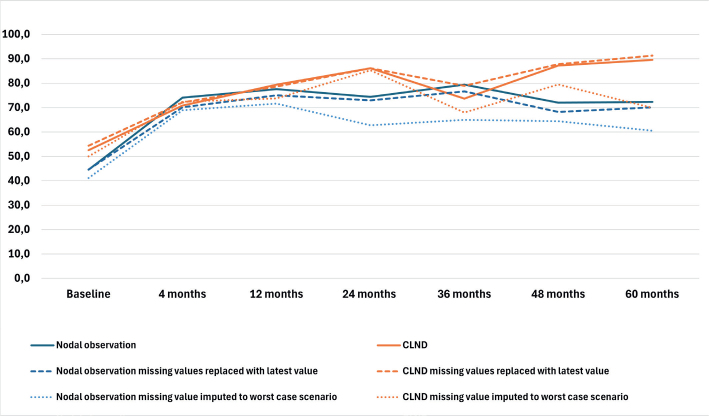
Role limitations due to physical health mean scores.

**Figure 4:**
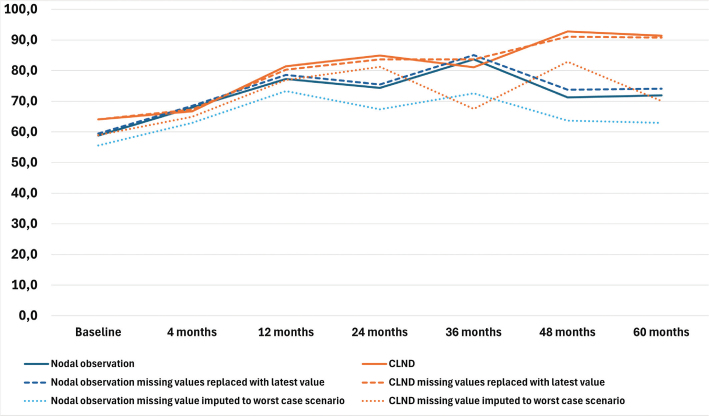
Role limitations due to emotional problems mean scores.

The results of the four RAND-36 subscales are presented in Figures 1–4 and in Table A in Supplementary Appendix. Initial analyses revealed statistically significant differences for three of the four subscales at 60 months, as well as one subscale at 48 months. After adjusting for multiple (28) comparisons using the FDR, the differences did not reach the significance threshold. At 60 months, the mean HRQoL score for the CLND and observation groups for General Health were 77.3 versus 65.0 (*p* = 0.007, adjusted *p* = 0.065), for Role limitations due to physical health 89.5 versus 72.3 (*p* = 0.029, adjusted *p* = 0.203) and for Role limitations due to emotional problems 91.4 versus 71.9 (*p* = 0.006, adjusted *p* = 0.065). At 48 months, for Role limitations due to emotional problems, the respective mean scores were 92.8 versus 71.3 (*p* = 0.002, adjusted *p* = 0.056). All these differences were clinically significant.

To address the issue of non-respondents, a sensitivity analysis was conducted where non-responses were assigned the lowest possible HRQoL score (0), based on the assumption that non-responses were associated with the worst possible health state. Under the worst-case scenario, the HRQoL mean scores dropped in comparison to the base analysis. The subscales Role limitations due to emotional problems and Role limitations due to physical health showed similar trends over time, while the General health and Physical functioning scores in both groups showed a decline over time, contrasting with the improvements observed in the primary analysis. In a sensitivity analysis carried out by replacing missing values with the latest available HRQoL score of that subscale, the HRQoL scores and trends showed similar values and trends over time in comparison to the base analysis. The results are presented in Figures 1–4.

The results of the correlation analysis including the correlation matrix tables are presented in the Appendix. Spearman correlations revealed statistically-significant moderate-to-strong positive correlations between subscales across time points. At 60 months the highest correlation was observed between Role limitations due to physical health and Role limitations due to emotional problems (Spearman’s rho 0.684 [95% CI 0.527–0.795], *p* < 0.001).

### Costs of care

**Figure 5:**
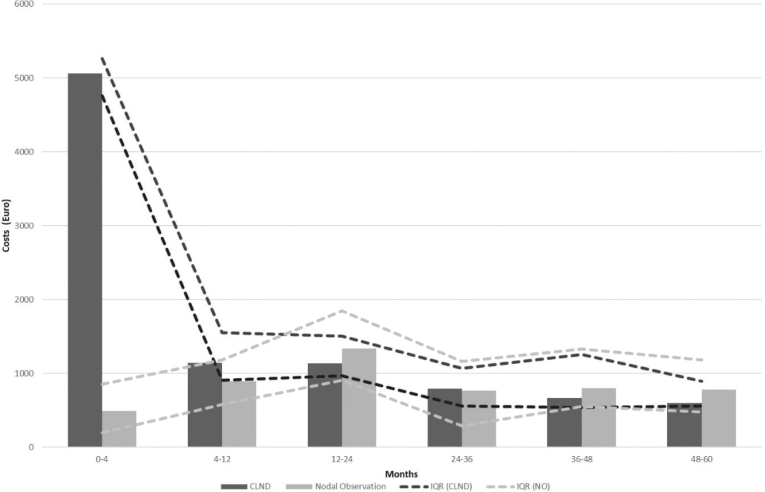
Median costs and IQR*. *IQR: interquartile range

Results of the cost comparison are presented in Figure 5. Median costs per patient were higher in the CLND group at 4 months but the difference disappeared during follow-up. The median cost per patient was slightly higher for the observation group at both 48 and 60 months, while there was no difference in the median cost. To address the issue of missing cost data, we performed a sensitivity analysis by imputing missing costs to 10% higher and lower. The costs did not change significantly in the sensitivity analysis (see Appendix Figure 1).

**Figure 6:**
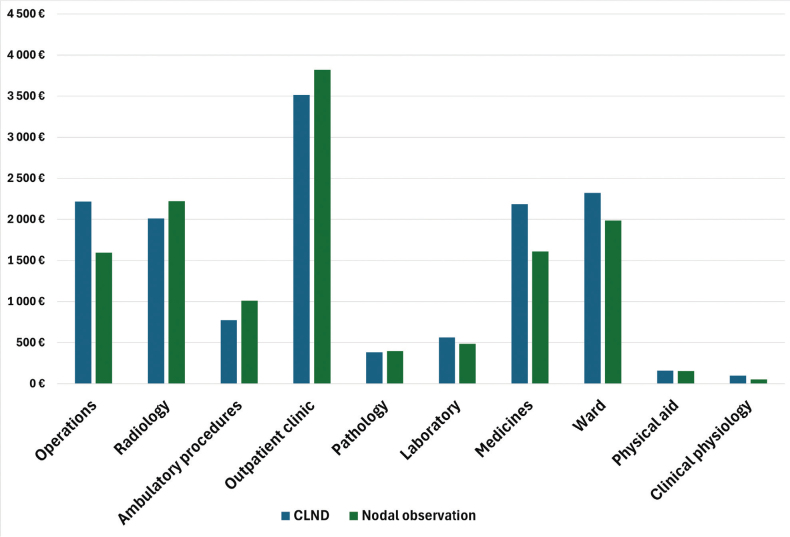
Distribution of costs per patient.

Total melanoma–related health care costs per patient from 1 January 2009 to 31 December 2019 were €13328 for the observation group and €14184 for the CLND group, excluding the cost of cancer therapies and drugs. The distribution of costs between the two groups are presented in Figure 6. Costs of complications per patient were €483,8 in the observation group versus €1143,5 in the CLND group.

## Discussion

While in the past several studies have examined morbidity among patients undergoing CLND versus SLNB alone [5, 14, 15], few have focused on HRQoL as the primary outcome. The aim of this study was to assess whether any increased morbidity associated with CLND had an effect on HRQoL and costs of care.

Surgical complications and morbidity are known to be frequent among patients undergoing CLND [5, 16]. In this study, 48.1% of the CLND patients had a complication at the CLND operation site, and both lymphedema prevalence and cumulative incidence were higher in the CLND group. Patient-reported swelling of the operated extremity was also higher among CLND patients throughout the study (Table 1). However, this difference in complications and surgery-related morbidity was not reflected as worse HRQoL among the CLND group.

Prior research using different instruments for assessing HRQoL suggests that the presence of patient-reported edema of the operated extremity, rather than dissection status, is a predictor of worse HRQoL [17, 18]. However, mild lymphedema may not cause symptoms and thus may not translate into worse HRQoL. Secondly, the instrument or subscales used may not be suitable to assess the effect of lymphedema on HRQoL. There may also have been some over reporting of lymphedema in the Finnish cohort. The diagnostic criteria for lymphedema in the trial protocol were intentionally not stringent to avoid underdiagnosis, to allow for some variation between study sites in recording and reporting mild lymphedema cases. The rates of lymphedema recorded for the trial overall were 24% for the CLND group and 6.3% for the observation group after a median follow-up of 43 months [1]. The follow-up-time for the Finnish population was slightly longer, but likely accounts for only a small increase in the cumulative incidence rate. The difference in lymphedema rates between the whole trial population and the Finnish patient cohort may also be partly due to confounding factors, such as swelling of the operated limb from other causes, higher BMI, certain medications or venous insufficiency, as well as differences in patient perceptions, culture and healthcare setting. The use of compression garments was not systematically recorded. 21.5% of the Finnish patients received nodal radiotherapy. The use of adjuvant nodal radiotherapy was not recorded in the MSLT-II trial, but differences in radiotherapy usage and protocols between centers could account for some of the difference.

In general, over time, the HRQoL measured by all subscales for both groups continued to improve up to 36 months. After 36 months, the HRQoL in the nodal observation groups plateaued or deteriorated, while the HRQoL of the CLND group continued to improve. The latter finding could be explained by several factors. Firstly, patient-reported swelling in the limb on the operated side was highest during the first months after surgery in both groups. In the CLND group, both the proportion of patients reporting swelling on the operated side and clinically diagnosed lymphedema prevalence continued to diminish after 36 months, while in the nodal observation group the percentage of patients reporting swelling increased slightly. Secondly, it can be hypothesized that the patients undergoing more extensive surgery estimate their HRQoL as being higher in the long term than those who have undergone less extensive surgery. This difference has also been observed when comparing the HRQoL of breast cancer patients undergoing immediate versus late breast reconstruction. Those undergoing late breast reconstruction are more satisfied after undergoing reconstruction in the long term, having lived in a worse health state before reconstructive surgery [19]. There may also be several other factors contributing to the HRQoL of melanoma patients, such as age, presence of metastatic disease, systemic therapies or, especially at the outset, the distress of receiving a cancer diagnosis and requiring treatment [20, 21, 22]. The two randomized groups in this study were similar in terms of age, cancer recurrence and the distribution of patients receiving systemic therapies, and this may be a reason why there were no major differences in HRQoL between the groups. The highest HRQoL scores were observed at 4 and 5 years among melanoma survivors, when the effects of treatments or the distress of newly-diagnosed cancer are likely to have been minimal. Another reason for the observation group scoring lower might be the distress and fear of disease progression or recurrence, since at the time of the trial it was still uncertain whether preforming immediate CLND might have a beneficial effect on survival. Prior research suggests that radiological imaging does not have a negative impact on HRQoL in the short term [22], but in the longer term it may increase the fear of disease recurrence. It should also be noted that while in the CLND group, four patients died between 36 and 60 months, in the observation group only one patient died during this period. The improvement in HRQoL among the CLND patients may thus partly be due to survival bias, though the small sample size limited further assessment of its effect.

While the results of this study do not conflict with existing evidence, there were several limitations which may have affected the interpretation and validity of the findings. The questionnaire used to evaluate the HRQoL of the MSLT-II patients was not standardized or validated and included only parts of the standardized and validated RAND-36 questionnaire. Thus, only four subscales of the RAND-36 could be utilized. This does not give a comprehensive picture of the HRQoL of the patients. The RAND-36 is also a generic HRQoL questionnaire and was not specifically developed for melanoma patients. Ideally, two different HRQoL questionnaires, one generic and one cancer-specific or melanoma-specific, would have been used.

The study was also limited by its relatively-small sample size, which may have reduced the power to detect statistically-significant differences, particularly after adjusting for multiple comparisons. In fact, the differences in HRQoL between the CLND and observation groups were not statistically significant after FDR correction. Additionally, the reduced sample size at 60 months, due to attrition, may have decreased statistical power.

The effect of missing HRQoL data should also be considered. The association between response status and survival at 5 years suggests answers may have not been missing at random. The worst-case scenario sensitivity analysis did not lead to major changes in mean HRQoL scores from baseline to 24 months, but from 36 months onwards the HRQoL deteriorated in both groups. However, this scenario is likely to overestimate the decline in HRQoL scores among the non-respondents. In the second sensitivity analysis, where missing values were replaced by the mean HRQoL scores at previous follow-up points, the scores did not change significantly and showed similar trends over time in comparison to the baseline analysis. This suggests that the results were not greatly affected by missing HRQoL data.

The patients also completed a 47-item HRQoL questionnaire (Supplementary Appendix). Only the information on patient-perceived edema was utilized for this study, where we wanted to focus on a standardized and validated HRQoL instrument. Future analysis and validation of the 47-item HRQoL instrument will provide insight into the validity of the results of this study.

The impact of missing cost data should also be considered when interpreting our findings. Our cost data included only tertiary care and thus most hospital costs. No details of the costs of primary care, home health care and out-patient medications or of indirect costs were available. However, since sentinel-node positive melanoma patients are followed up and treated in tertiary health care facilities and thus, melanoma-related costs are generated in tertiary care, while primary health care services are, in general, lower in cost. Some higher primary health-care costs, especially costs of end-of life care at primary health care facilities may have been missing, but the differences in these costs between the two groups were likely minimal, since the two groups did not differ in cancer progression or survival.

Cost data before 2009 were imputed using the median imputation method based on cost data after 2009. This causes some uncertainty and possible bias in measurements taken at 4 months. However, the sensitivity analysis did not lead to major changes in the cost analysis and therefore our analysis seems to be robust despite missing cost data. In the nodal observation group, the proportion of missing data was so small that a 10% discount or increase in the missing costs did not change the median costs or interquartile range. It should nevertheless be noted that our cost data were gathered during a RCT and may not accurately reflect the true costs of care in a non-trial setting.

While MSLT-II and DeCOG-SLT demonstrated that no significant survival benefit was achieved by performing immediate CLND instead of nodal observation, our results suggest that there may be no major differences in HRQoL between patients undergoing immediate CLND or nodal observation. For this study, we examined only the Finnish patients, which resulted in a small sample size. While analysis of HRQoL for the whole MSLT-II population could confirm the observed effects and assess their robustness and clinical relevance, it is generally recommended that HRQoL data should be gathered separately from different countries, especially when combined with a cost-analysis, to better account for the effects of differing cultures, healthcare systems and social factors [23, 24]. To further validate the results of the HRQoL assessment of this study and to overcome the issue of small sample size, future studies comparing the HRQoL data from other MSLT-II centers as well as real-life long-term cost data from the CLND and nodal observation groups could provide further insight into possible differences in cost-effectiveness of these two management strategies.

## Supplementary Material

Health-related quality of life and hospital costs of Finnish melanoma patients participating in the second Multicenter Selective Lymphadenectomy Trial

## Data Availability

The data used in this study contain sensitive patient information and cannot be made public for legislative reasons.
