# Supplementary material for: Health-related quality of life and hospital costs of Finnish melanoma patients participating in the second Multicenter Selective Lymphadenectomy Trial
Source: Acta Oncol. 2025 Feb 10;64:42314. doi: 10.2340/1651-226X.2025.42314 (PMC11833325; doi:10.2340/1651-226X.2025.42314)
Supplement: Health-related quality of life and hospital costs of Finnish melanoma patients participating in the second Multicenter Selective Lymphadenectomy Trial [file AO-64-42314-s1.pdf]

Supplementary Figure 1. Costs of care, sensitivity analysis.

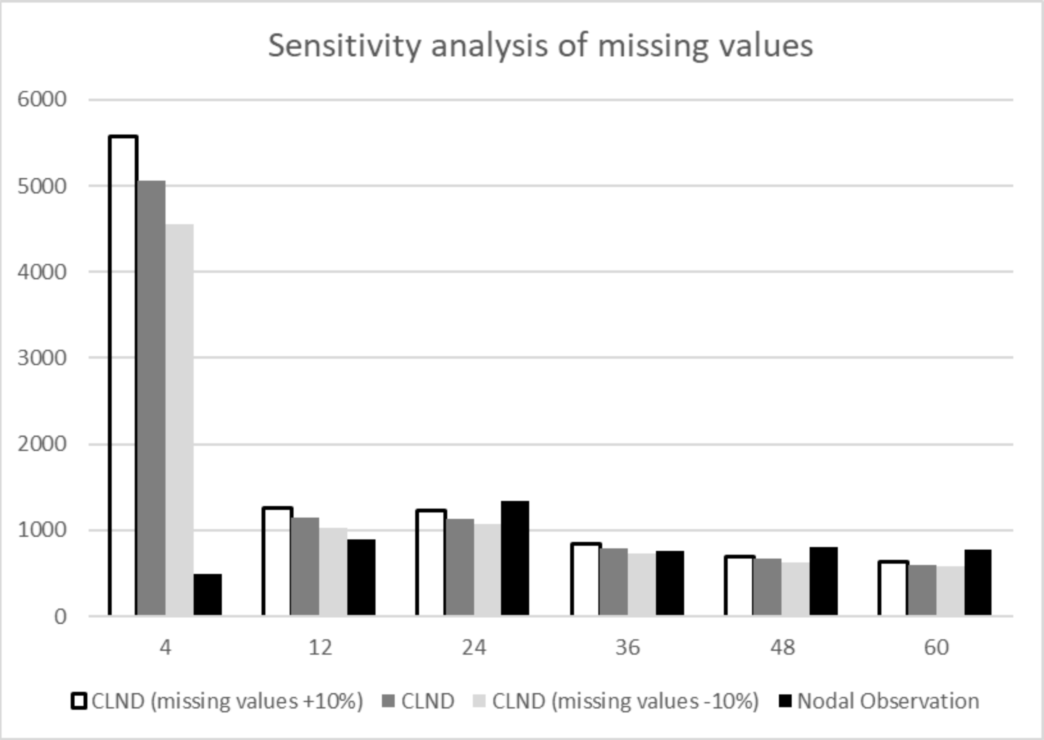

Supplementary Appendix

The corresponding RAND-36/ MOS Core Survey Questions and MOS Health Survey questions.

1. MOS -> 1. RAND-36
2. MOS -> 2. RAND-36
3. a. MOS -> 3. RAND-36
   b. MOS -> 4. RAND-36
   c. MOS -> 5. RAND-36
   d. MOS -> 6. RAND-36
   e. MOS-> 7. RAND-36
   f. MOS –> 8. RAND-36
   g. MOS -> 9. RAND-36
   h. MOS -> 10. RAND-36
   i. MOS -> 11. RAND-36
   j. MOS -> 12. RAND-36
4. a. MOS -> 13. RAND-36
   b. MOS -> 14. RAND-36
   c. MOS -> 15. RAND-36
   d. MOS -> 16. RAND-36
5. a. MOS -> 17. RAND-36
   b. MOS -> 18. RAND-36
   c. MOS -> 19. RAND-36
6. a. MOS -> 33. RAND-36
   b. MOS -> 34. RAND-36
   c. MOS -> 35. RAND-36
   d. MOS -> 36. RAND-36
7. a. MOS - > 30. MOS Core Survey
   b. MOS -> 33. MOS Core Survey
   c. MOS -> 24. MOS Core Survey
   d. MOS -> 53. MOS Core Survey
   e. MOS -> 54. MOS Core Survey

Supplementary Table A. HRQoL results

Dimension	Time point	Dissection group mean (median)	IQR	Nodal observation mean (median)	IQR	p-value (Mann-Whitney-U-test)	Benjamini-Hochberg FDR adjusted p-value
General Health	Baseline	71.3 (75.0)	20.0	66.4 (70.0)	25.0	0.058	0.325
	4 months	66.4 (67.5)	25.0	68.9 (70.0)	20.0	0.477	0.837
	12 months	71.4 (70.0)	25.0	67.7 (18.8)	27.5	0.278	0.730
	24 months	70.9 (75.0)	30.0	66.1 (70.0)	30.0	0.339	0.730
	36 months	68.5 (70.0)	27.5	69.0 (75.0)	20.0	0.954	0.994
	48 months	73.1 (75.0)	23.8	64.6 (65.0)	31.3	0.077	0.359
	60 months	77.3 (80.0)	30.0	65.0 (65.0)	22.5	0.007*	0.065
Physical Functioning	Baseline	75.0 (80.0)	35.0	77.6 (85.0)	35.0	0.668	0.855
	4 months	83.5 (90.0)	30.0	85.1 (95.0)	20.0	0.508	0.837
	12 months	88.1 (90.0)	20.0	84.9 (90.0)	25.0	0.625	0.855
	24 months	86.2 (90.0)	25.0	87.7 (95.0)	17.5	0.670	0.855
	36 months	85.5 (95.0)	22.5	88.6 (85.0)	15.0	0.702	0.855
	48 months	88.9 (95.0)	15.0	84.4 (95.0)	21.3	0.283	0.730
	60 months	87.4 (95.0)	20.0	85.3 (95.0)	25.0	0.749	0.874
Role limitations due to physical health	Baseline	52.5 (50.0)	100.0	44.4 (25.0)	100.0	0.317	0.730
	4 months	70.8 (100.0)	68.8	74.0 (100.0)	50.0	0.994	0.994
	12 months	79.4 (100.0)	25.0	77.6 (100.0)	37.5	0.455	0.837
	24 months	86.1 (100.0)	25.0	74.4 (100.0)	50.0	0.119	0.476
	36 months	73.7 (100.0)	75.0	79.4 (100.0)	25.0	0.698	0.855
	48 months	87.2 (100.0)	25.0	72.0 (100.0)	56.3	0.176	0.616
	60 months	89.5 (100.0)	0.0	72.3 (100.0)	62.5	0.029*	0.203
Role limitations due to emotional problems	Baseline	64.1 (66.7)	66.7	58.8 (66.7)	100.0	0.636	0.855
	4 months	66.7 (100.0)	66.7	68.0 (66.7)	66.7	0.959	0.994
	12 months	81.4 (100.0)	33.3	77.3 (100.0)	33.3	0.498	0.867

	24 months	84.9 (100.0)	33.3	74.4 (100.0)	33.3	0.332	0.730
	36 months	81.1 (100.0)	33.3	83.7 (100.0)	33.3	0.860	0.963
	48 months	92.8 (100.0)	0.0	71.3 (100.0)	66.7	0.002*	0.056
	60 months	91.4 (100.0)	0.0	71.9 100.0)	66.7	0.006*	0.065

* significant at 0.05 level

IQR = interquartile range

Supplementary table. Correlation Matrix at baseline

		Physical functioning at baseline (95%CI)	Role limitations due to physical health at baseline (95%CI)	Role limitations due to emotional problems at baseline (95%CI)	General health at baseline (95%CI)
Spearman's rho	Physical functioning at baseline	1.000	0.604 (0.461 – 0.717)	0.403 (0.223 – 0.557)	0.369 (0.186 – 0.528)
	Significance (2-tailed)		< 0.001**	< 0.001**	< 0.001**
	N	105	103	104	105
	Role limitations due to physical health at baseline	0.604 (0.461 – 0.717)	1.000	0.517 (0.355 – 0.649)	0.453 (0.281 – 0.597)
	Significance (2-tailed)	<0.001**		<0.001**	<0.001**
	N	103	105	104	105
	Role limitations due to emotional problems at baseline	0.403 (0.223 – 0.557)	0.517 (0.355 – 0.649)	1.000	0.397 (0.218 – 0.550)
	Significance (2-tailed)	<0.001**	<0.001**		<0.001**
	N	104	104	106	106
	General health at baseline	0.369 (0.186 – 0.528)	0.453 (0.281 – 0.597)	0.397 (0.218 – 0.550)	1.000
	Significance (2-tailed)	<0.001**	<0.001**	<0.001**	
	N	105	105	106	107

** Correlation significant at the < 0.001 level (2-tailed).

Supplementary table. Correlation Matrix at 4 months.

		Physical functioning at 4 months (95% CI)	Role limitations due to physical health at 4 months (95% CI)	Role limitations due to emotional problems at 4 months (95% CI)	General health at 4 months (95% CI)
Spearman's rho	Physical functioning at 4 months	1.000	0.627 (0.483 – 0.738)	0.414 (0.229 – 0.570)	0.570 (0.412 – 0.694)
	Significance (2-tailed)		<0.001**	<0.001**	<0.001**
	N	98	96	98	96
	Role limitations due to physical health at 4 months	0.627 (0.483 – 0.738)	1.000	0.673 (0.543 – 0.771)	0.438 (0.255 – 0.591)
	Significance (2-tailed)	<0.001**		<0.001**	<0.001**
	N	96	99	98	96
	Role limitations due to emotional problems at 4 months	0.414 (0.229 – 0.570)	0.673 (0.543 – 0.771)	1.000	0.465 (0.288 – 0.611)
	Significance (2-tailed)	<0.001**	<0.001**		<0.001**
	N	98	98	100	98
	General health at 4 months	0.570 (0.412 – 0.694)	0.438 (0.255 – 0.591)	0.465 (0.288 – 0.611)	1.000
	Significance (2-tailed)	<0.001**	<0.001**	<0.001**	
	N	98	96	98	98

** Correlation significant at the < 0.001 level (2-tailed).

CI = Confidence interval

Supplenetary table. Correlation Matrix at 12 months.

		Physical functioning at 12 months (95% CI)	Role limitations due to physical health at 12 months (95% CI)	Role limitations due to emotional problems at 12 months (95% CI)	General health at 12 months (95% CI)
Spearman's rho	Physical functioning at 12 months	1.000	0.569 (0.402 – 0.698)	0.344 (0.142 – 0.519)	0.467 (0.282 – 0.618)
	Significance (2-tailed)		<0.001**	<0.001**	<0.001**
	N	91	88	90	90
	Role limitations due to physical health at 12 months	0.569 (0.402 – 0.698)	1.000	0.669 (0.530 – 0.772)	0.563 (0.397 – 0.694)
	Significance (2-tailed)	<0.001**		<0.001**	<0.001**
	N	88	89	89	89
	Role limitations due to emotional problems at 12 months	0.344 (0.142 – 0.519)	0.669 (0.530 – 0.772)	1.000	0.544 (0.376 – 0.678)
	Significance (2-tailed)	<0.001**	<0.001**		<0.001**
	N	90	89	93	91
	General health at 12 months	0.467 (0.282 – 0.618)	0.563 (0.397 – 0.694)	0.544 (0.376 – 0.678)	1.000
	Significance (2-tailed)	<0.001**	<0.001**	<0.001**	
	N	90	89	91	92

** Correlation significant at the < 0.001 level (2-tailed).

CI= confidence interval

Supplementary table. Correlation Matrix at 24 months.

		Physical functioning at 24 months (95% CI)	Role limitations due to physical health at 24 months (95% CI)	Role limitations due to emotional problems at 24 months (95% CI)	General health at 24months (95% CI)
Spearman's rho	Physical functioning at 24 months	1.000	0.541 (0.363 – 0.681)	0.257 (0.037 – 0.352)	0.534 (0.354 – 0.676)
	Significance (2-tailed)		<0.001**	0.019*	<0.001**
	N	84	83	83	83
	Role limitations due to physical health at 24 months	0.541 (0.363 – 0.681)	1.000	0.426 (0.226 – 0.592)	0.606 (0.445 – 0.729)
	Significance (2-tailed)	<0.001**		<0.001**	<0.001**
	N	83	84	83	84
	Role limitations due to emotional problems at 24 months	0.257 (0.037 – 0.352)	0.426 (0.226 – 0.592)	1.000	0.419 (0.217 – 0.586)
	Significance (2-tailed)	0.019*	<0.001	1.000	<0.001
	N	83	83	85	83
	General health at 24 months	0.534 (0.354 – 0.676)	0.606 (0.445 – 0.729)	0.419 (0.217 – 0.586)	1.000
	Significance (2-tailed)	<0.001**	<0.001**	<0.001**	
	N	83	84	83	84

** Correlation significant at the 0.001 level (2-tailed). * Correlation significant at the 0.05 level (2-tailed).

CI = Confidence interval

Supplementary table. Correlation Matrix at 36 months.

		Physical functioning at 36 months (95% CI)	Role limitations due to physical health at 36 months (95% CI)	Role limitations due to emotional problems at 36 months (95% CI)	General health at 36months (95% CI)
Spearman's rho	Physical functioning at 36 months	1.000	0.706 (0.567 – 0.806)	0.438 (0.229 – 0.608)	0.640 (0.477 – 0.761)
	Significance (2-tailed)		<0.001**	<0.001**	<0.001
	N	76	76	76	74
	Role limitations due to physical health at 36 months	0.706 (0.567 – 0.806)	1.000	0.638 (0.478 – 0.757)	0.579 (0.401 – 0.715)
	Significance (2-tailed)	<0.001**		<0.001**	<0.001**
	N	76	78	77	76
	Role limitations due to emotional problems at 36 months	0.438 (0.229 – 0.608)	0.638 (0.478 – 0.757)	1.000	0.456 (0.249 – 0.623)
	Significance (2-tailed)	<0.001**	<0.001		<0.001**
	N	76	77	78	75
	General health at 36 months	0.640 (0.477 – 0.761)	0.579 (0.401 – 0.715)	0.456 (0.249 – 0.623)	1.000
	Significance (2-tailed)	<0.001**	<0.001**	<0.001**	
	N	74	76	75	76

** Correlation significant at the < 0.001 level (2-tailed).

CI = Confidence interval

Supplementary table. Correlation Matrix at 48 months.

		Physical functioning at 48 months (95% CI)	Role limitations due to physical health at 48 months (95% CI)	Role limitations due to emotional problems at 48 months (95% CI)	General health at 48 months (95% CI)
Spearman's rho	Physical functioning at 48 months	1.000	0.698 (0.559 – 0.799)	0.421 (0.214 – 0.692)	0.670 (0.520 – 0.780)
	Significance (2-tailed)		<0.001**	<0.001**	<0.001**
	N	79	78	79	77
	Role limitations due to physical health at 48 months	0.698 (0.559 – 0.799)	1.000	0.382 (0.169 – 0.560)	0.589 (0.415 – 0.722)
	Significance (2-tailed)	<0.001**		<0.001**	<0.001**
	N	78	79	79	77
	Role limitations due to emotional problems at 48 months	0.421 (0.214 – 0.692)	0.382 (0.169 – 0.560)	1.000	0.397 (0.185 – 0.574)
	Significance (2-tailed)	<0.001	<0.001		<0.001
	N	79	79	80	78
	General health at 48 months	0.670 (0.520 – 0.780)	0.589 (0.415 – 0.722)	0.397 (0.185 – 0.574)	1.000
	Significance (2-tailed)	<0.001**	<0.001**	<0.001**	
	N	77	77	78	78

** Correlation significant at the < 0.001 level (2-tailed).

CI = Confidence Interval

Supplementary table. Correlation Matrix at 60 months.

		Physical functioning at 60 months (95% CI)	Role limitations due to physical health at 60 months (95% CI)	Role limitations due to emotional problems at 60 months (95% CI)	General health at 60 months (95% CI)
Spearman's rho	Physical functioning at 60 months	1.000	0.587 (0.399 – 0.727)	0.384 (0.155 – 0.574)	0.674 (0.513 – 0.789)
	Significance (2-tailed)		<0.001**	<0.001**	<0.001**
	N	69	68	69	68
	Role limitations due to physical health at 60 months	0.587 (0.399 – 0.727)	1.000	0.684 (0.527 – 0.795)	0.490 (0.277 – 0.658)
	Significance (2-tailed)	<0.001**		<0.001**	<0.001**
	N	68	68	68	67
	Role limitations due to emotional problems at 60 months	0.384 (0.155 – 0.574)	0.684 (0.527 – 0.795)	1.000	0.445 (0.224 – 0.622)
	Significance (2-tailed)	0.001**	<0.001**		<0.001**
	N	69	68	69	68
	General health at 60 months	0.674 (0.513 – 0.789)	0.490 (0.277 – 0.658)	0.445 (0.224 – 0.622)	1.000
	Significance (2-tailed)	<0.001**	<0.001**	<0.001**	
	N	68	67	68	68

** Correlation significant at the <0.001 level (2-tailed).

CI = Confidence Interval
